# Role of housing in blood pressure control: a review of evidence from the Smart Wellness Housing survey in Japan

**DOI:** 10.1038/s41440-022-01060-6

**Published:** 2022-10-13

**Authors:** Wataru Umishio, Toshiharu Ikaga, Kazuomi Kario, Yoshihisa Fujino, Masaru Suzuki, Shintaro Ando, Tanji Hoshi, Takesumi Yoshimura, Hiroshi Yoshino, Shuzo Murakami, on behalf of the SWH survey group

**Affiliations:** 1 Department of Architecture and Building Engineering, School of Environment and Society, Tokyo Institute of Technology, Ookayama, Meguro-ku, Tokyo, Japan; 2 Department of System Design Engineering, Faculty of Science and Technology, Keio University, Yokohama, Kanagawa Japan; 3 Department of Cardiology, Jichi Medical University School of Medicine, Shimotsuke, Tochigi Japan; 4 Department of Environmental Epidemiology, Institute of Industrial Ecological Sciences, University of Occupational and Environmental Health, Kitakyushu, Fukuoka Japan; 5 Department of Emergency Medicine, Ichikawa General Hospital, Tokyo Dental College, Ichikawa, Chiba Japan; 6 Department of Architecture, Faculty of Environmental Engineering, The University of Kitakyushu, Kitakyushu, Fukuoka Japan; 7 Tokyo Metropolitan University, Hachioji, Tokyo, Japan; 8 University of Occupational and Environmental Health, Kitakyushu, Fukuoka Japan; 9 Tohoku University, Sendai, Miyagi Japan; 10 Institute for Built Environment and Carbon Neutral for SDGs, Hirakawacho, Chiyoda-ku, Tokyo, Japan

**Keywords:** Blood pressure variability, Home blood pressure, Housing, Indoor temperature, Insulation retrofit

## Abstract

Current countermeasures for preventing hypertension emphasize only improvements to lifestyle. Recently, improving life environment has attracted attention, in parallel with publication of the WHO Housing and health guidelines. We quantitatively evaluated the relationship between housing thermal environment and blood pressure (BP) in a real-world setting. We conducted a nationwide, prospective intervention study—the Smart Wellness Housing survey—in Japan, as a non-randomized controlled trial. The intervention was the retrofitting of thermal insulation in houses. Participant recruitment was done by construction companies in all 47 prefectures of Japan. Measurements of home BP and indoor temperature at 1.0 m above the floor in the living room, changing room, and bedroom were taken for 2 weeks before and after the intervention each winter (November–March) of FY 2014–2019. As of July 2022, over 2500 households and 5000 participants were registered in the database. We found that (1) about 90% of Japanese lived in cold homes (minimum indoor temperature <18 °C), (2) indoor temperature was non-linearly associated with home BP, (3) morning systolic BP (SBP) was more sensitive than evening SBP to changes in indoor temperature, (4) SBP was influenced by indoor temperature change particularly in older participants and women, (5) unstable indoor temperature was associated with large BP variability, and (6) insulation retrofitting intervention significantly reduced home BP, especially in hypertensive patients. We proposed that the BP reduction effect of the life-environment is comparable to that achievable by lifestyle.

## Abstract (web summary)

Hypertension and cardiovascular diseases are not only lifestyle diseases but also life-environment diseases.

## Graphical abstract

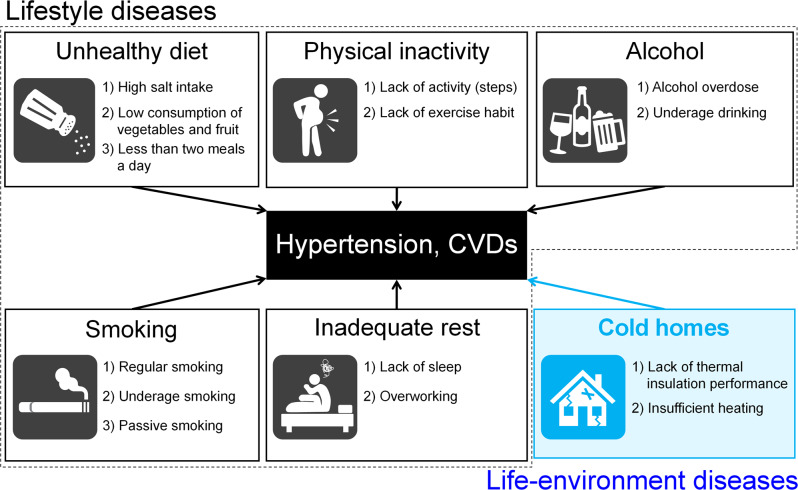

## Introduction

Excess winter mortality (EWM), which refers to the marked increase in mortality rate in winter, is a global problem in public health [1, 2]. Paradoxically, however, studies in Europe [3–5], the USA [6, 7], and Asia [8, 9] have reported higher EWM in areas with milder winter climates. One possible explanation for this is that houses in these areas are less adequately prepared for winter conditions. More than half of EWM is caused by cardiovascular disease (CVD) [10], a phenomenon which is partially attributable to cold-induced hypertension.

Hypertension is called a “silent killer” as it has almost no subjective symptoms [11]. Indeed, many hypertensive patients are unaware of their hypertension [12]. Reliance on antihypertensive drugs alone might be an inadequate, high-risk strategy—a likely better strategy would involve shifting the BP of the total population in the desirable direction. However, existing population strategies exclusively emphasize improvements to lifestyle, focusing on habits such as physical activity, diet, smoking and alcohol consumption; improving the housing thermal environment has been neglected.

In 2018, the WHO issued the Housing and health guidelines, which focused on “low indoor temperatures and insulation” [13]. Based on a systematic review of evidence the guidelines recommend a minimum indoor temperature of 18 °C, stating that low indoor temperature can lead to vasoconstriction—a known risk factor of hypertension—and that retrofitting insulation into existing housing can alleviate the negative effects of low indoor temperature on health. The guidelines call for research to establish the appropriateness of 18 °C as a general target for a minimum indoor temperature or whether this target should vary in different populations. The guidelines also emphasize the desirability of further evidence on the effects of living in a thermally insulated home on health outcomes.

Consistent with the above background, we started a nationwide, prospective intervention trial, the “Smart Wellness Housing (SWH) survey” in Japan, to quantitatively evaluate the health effects of indoor temperature and insulation retrofitting of houses.

### Study design of the Smart Wellness Housing survey

**Fig. 1:**
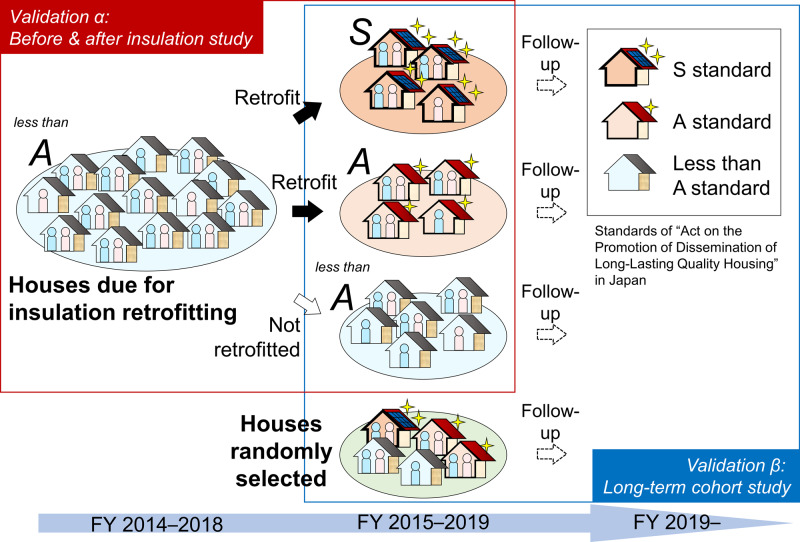
Overview of the Smart Wellness Housing survey in Japan †FY indicates fiscal year

The SWH survey was designed with two validation patterns, prepared from short- and long-term viewpoints (Fig. 1). The survey started in winter 2014, and collected all data used in the “before and after insulation study” described below. Construction companies recruited participants throughout all 47 prefectures of Japan, and more than 2500 households and 5000 participants were registered in the SWH survey database as of July 2022.

1. Before and after insulation study [University Hospital Medical Information
  Network Clinical Trials Registry (UMIN-CTR) Trial No. UMIN000030601]
  This study was a non-randomized controlled trial with groups categorized according to participants’ choice of whether or not to conduct insulation retrofitting. This intervention included a range of treatments, including heat-insulation work on the outer walls, floor and/or roof; replacing single-glazed with double-glazed windows; and replacing window frames. Thermal insulation performance level after retrofitting was set to meet the S standards (approximately equivalent to a long-life newly built, high-quality house) or A standards (lower than S standards but expected to provide consistently improved performance) of the ‘Act on the Promotion of Dissemination of Long-Lasting Quality Housing’ in Japan [14]. This study investigated short-term changes in indoor environment and participants’ health pre- and post-insulation retrofitting.
2. Long-term cohort study (UMIN-CTR Trial No. UMIN000042196)

This cohort study aims to evaluate long-term health effects in relation to differences in thermal insulation level. It is being conducted by a follow-up survey of households for more than 5 years after the completion of insulation retrofitting. Houses with no insulation retrofitting were randomly selected and included as a control group.

Participants measured the indoor thermal environment and their home blood pressure (HBP), and kept a diary for 2 weeks, mainly during winter (November–March). They also responded to a questionnaire during the same period. Indoor temperature and relative humidity at 1.0 m above the floor were monitored in the living room, changing room, and bedroom at 10-min intervals by automated monitoring sensors supplied by the investigators and installed by the participants. HBP was also measured twice in the morning and twice in the evening, in accordance with the Japanese guideline [15]. Health checkup data [e.g., blood lipids, blood glucose, and electrocardiogram (ECG)] were also collected whenever possible.

### Indoor temperature in Japan and high-risk residents

Japan stretches across several climate zones from north to south. The climate is mostly temperate, but becomes subarctic mainly in Hokkaido. According to data from the Ministry of Land, Infrastructure, Transport and Tourism, 30% of the ~50 million houses in Japan are without insulation, and in 2018, only 11% were sufficiently insulated to meet the country’s highest thermal insulation standards [16]. Furthermore, in contrast to the continuous heating of entire buildings typical in Europe and the USA, intermittent heating of the living room only is the general practice in Japan. Therefore, there is concern that indoor temperatures in Japan may be lower than in European and American countries.

**Fig. 2:**
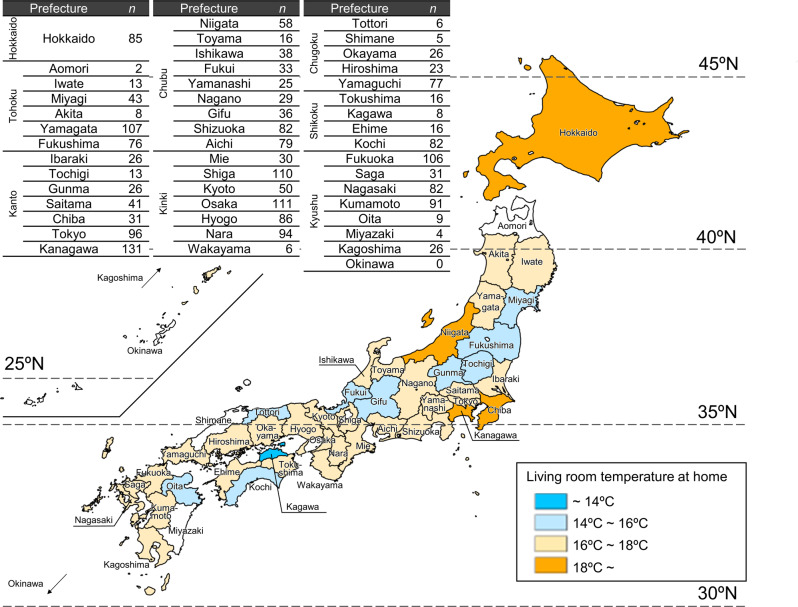
Average living room temperature at home in each prefecture from ref. [17] †Excluding prefectures with data from 5 participating households or less (displayed in white)

We reported the actual status of houses throughout Japan before insulation retrofitting [17]. Cross-sectional analyses involving 2190 houses revealed average temperatures when participants were at home in the living room and changing room of 16.8 °C and 13.0 °C, respectively, and 12.8 °C when participants slept in the bedroom. Minimum temperatures in the living room, changing room, and bedroom were 12.6 °C, 10.4 °C, and 11.2 °C, respectively. Minimum temperatures were below the 18 °C recommended in the WHO guidelines in over 90% of households. A comparison of average living room temperatures across prefectures is shown in Fig. 2. The highest temperature (19.8 °C) was in Hokkaido, where the climate is severe and houses have higher thermal insulation criteria than in other areas. In contrast, the lowest average living room temperature (13.1 °C) was in Kagawa, with a mild climate. The maximum difference between prefectures was 6.7 °C.

We also examined characteristics common to residents of cold houses. Lower household income was a risk factor for living in cold houses, likely because it may force residents to use heating sparingly or to live in houses with poor insulation. Single-person household was also a risk factor. Furthermore, lower room temperature was associated with the use of a kotatsu (traditional Japanese heating device which does not heat an entire room) and wearing of multiple layers of clothes. These results will aid in identifying “high-risk” residents in greater need of active housing intervention.

Separately to our study, Saeki et al. reported that mean living room/bedroom temperature was 16.1/12.6 °C in the cold season (October–April) in the Kansai region [18], and Uchiyama et al. found that mean temperature in the living room in 602 houses throughout Japan was 17 °C during winter (January–March) [19], which was almost the same as our result. Elsewhere, French et al. collected indoor temperatures of 397 houses in New Zealand during the winter season and reported mean living room daytime temperatures and nighttime bedroom temperatures of 15.8 °C and 13.6 °C, respectively [20], similar to Japan. In contrast, a study which reviewed indoor temperatures in UK homes reported average winter living room temperatures of 18–21 °C [21], while the average winter living room temperature in New York apartments was 23.3 °C [22], indicating more comfortable conditions in Europe and the USA than Japan. However, even in European and American countries, fuel (or energy) poverty, defined as an energy cost of maintaining an adequate indoor temperature of more than 10% of household income, is a prevalent problem [23, 24]. Thus, we note that living in cold homes is an issue not only in Japan, but also in some other countries.

### Indoor temperature and blood pressure

As described above, we established that many Japanese people live in cold homes. In view of the concern that low indoor temperatures causes high blood pressure, we analyzed the association between HBP and indoor winter temperature using a multilevel model [25]. Cross-sectional analyses based on ~33,000 data points derived from 2900 residents revealed that HBP had a significant inverse association with indoor temperature: HBP was higher at low indoor temperatures. Of note, systolic blood pressure (SBP) was significantly more sensitive to changes in indoor temperature in the morning (8.2 mmHg increase/10 °C decrease) than in the evening (6.5 mmHg increase/10 °C decrease) in residents aged 57 years (mean age of participants in this survey). Because CVD-related crises occur frequently in the morning [26–29] and morning HBP strongly predicts cardiovascular events [30–35], this finding strengthens the importance of morning indoor temperature management in reducing the danger from CVDs.

**Fig. 3:**
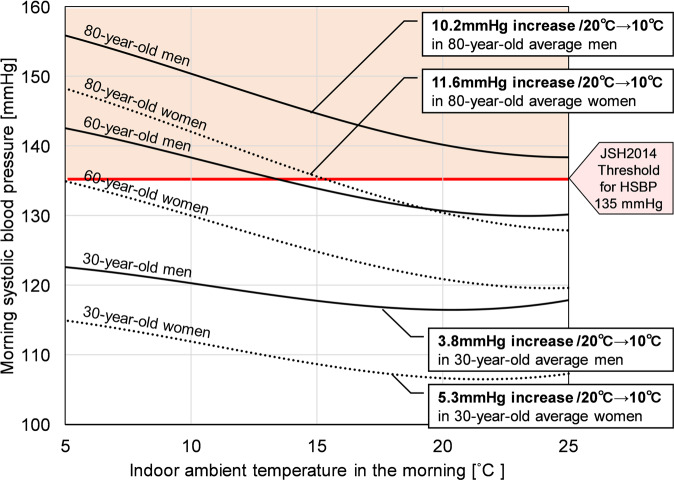
Relationship between indoor ambient temperature and morning systolic blood pressure from ref. [25] †Average values for male/female participants in the Smart Wellness Housing survey were inputted into the multilevel model in ref. [25]: vegetable consumption = regularly, exercise = rarely, current smoking status = nonsmoker, alcohol consumption = every day/none, antihypertensive drug use = none. JSH indicates Japanese Society of Hypertension; and HSBP, home systolic blood pressure

We found a nonlinear cubic relationship between morning SBP and indoor temperature, illustrated in Fig. 3. The relationship between HSBP and indoor temperature was weaker at low and high temperature ranges. This might be a consequence of thermoregulatory behaviors such as adding or removing clothes at low and high temperatures, and of limitations in thermophysiological reactions including vasoconstriction and vasodilatation. However, this evidence also highlights the importance of indoor temperature even if not extremely low, because SBP changes steeply at the middle temperature range. A previous systematic review and meta-analysis [36] did not determine optimum home temperature because it considered the temperature–BP relationship as a linear function. Therefore, we propose that the nonlinear temperature–BP relationship revealed in our study can contribute towards establishing optimum home temperature recommendations

We also found that SBP in older residents as well as women was particularly susceptible to indoor temperature change. Possible causes here are vascular endothelial dysfunction and diminished physiological adaptability (e.g., vasodilatation) to indoor temperature variations in older residents. Additionally, older residents and women have decreased muscle mass compared with younger residents and men, which translates to reduced metabolic heat production and increased vulnerability to cold. For these reasons, recommendations concerning optimum home temperature should be tailored according to population group, a topic identified as requiring future research in the WHO Housing and health guidelines.

Recently, Tai et al. [37] examined the relationship between ambulatory BP and skin temperature among 584 older adults, showing that the mediation effect of skin temperature and importance of distal skin temperature. This evidence promotes an understanding of the underlying thermophysiological mechanism and provides a clue of controlling BP.

### Indoor temperature instability and blood pressure variability

**Fig. 4:**
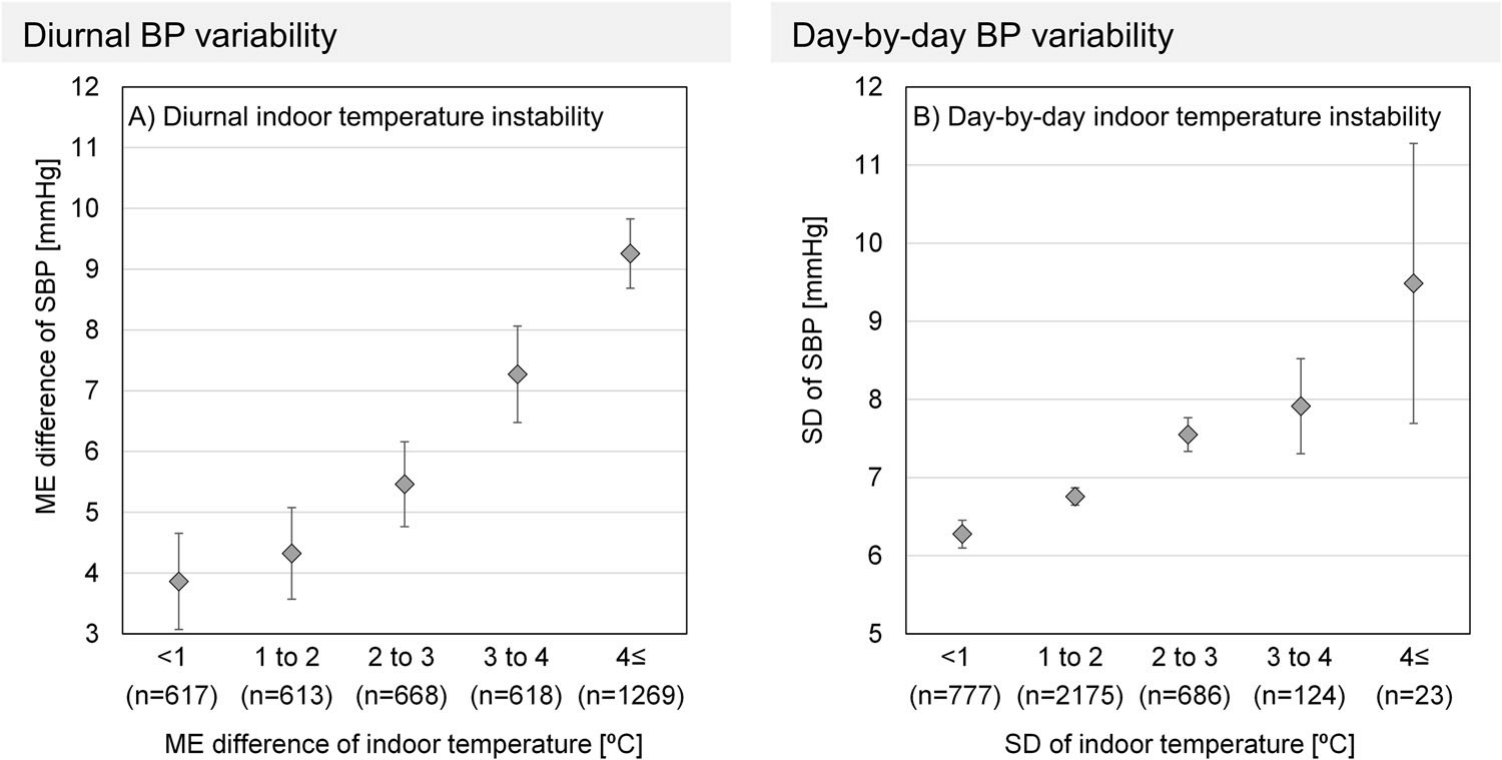
Relationship between blood pressure variability and indoor temperature instability re-edited from ref. [37] †A: morning-evening (ME) difference in systolic blood pressure (SBP) and ME difference in indoor temperature. B: standard deviation (SD) of SBP and SD of indoor temperature. The plot shows the average of each group, and the error bar shows the 95% confidence interval. SD was calculated from a 2-week measurement. ME average was used as a representing value of each day when calculating SD

In addition to BP level, BP variability requires due consideration when evaluating the risk of cardiovascular events. We hypothesized that a stable home thermal environment helps to reduce BP variability, and evaluated the indoor temperature–BP relationship from another perspective [38]. Over a 2-week period we used the morning-evening (ME) difference as an index of diurnal variability, and the standard deviation (SD), coefficient of variation (CV), variability independent of the mean (VIM) as well as average real variability (ARV) as indices of day-by-day variability. The mean ME difference in indoor/outdoor temperature (overnight decrease) was 3.2/1.5 °C, and the mean indoor/outdoor temperature SD was 1.6/2.5 °C. As shown in Fig. 4, compared to participants living in houses with an overnight indoor temperature decrease (ME difference in indoor temperature) of less than 1 °C, the ME difference in SBP was more than double in participants living in houses with an ME difference in indoor temperature ≥4 °C. Concerning day-by-day variability, compared to participants whose houses had an indoor temperature SD < 1 °C, the SD of SBP was larger in participants whose houses had an SD ≥ 4 °C. Linear regression analyses adjusted for confounders showed a strong correlation between the ME difference in indoor temperature and the ME difference in SBP. The indoor temperature SD also showed an association with the SBP SD. Trends for CV, VIM, and ARV were similar to BP SD. By contrast, instability in outdoor temperature showed an association with neither diurnal nor day-by-day HBP variability. Of interest, Nakagami et al. [39] evaluated the effect of houses on BP level and variability by comparing 24-h BP at home and at a highly insulated model house. They found that residents who usually lived in a cold house had lower BP and smaller BP variability when in the model house, where indoor temperature was high and stable. Given that Narita et al. [40] showed that day-by-day HBP variability is more strongly associated with future CVD events in winter than in other seasons, these findings show that winter residents should keep the indoor temperature not only warm but stable to reduce BP level and variability.

BP also shows long-term fluctuations [41, 42]; these include seasonal variation, which has been associated with cardiovascular events [43]. Several recent papers have summarized evidence on seasonal BP variation [44, 45], one of which recommended optimizing environmental factors such as room temperature and housing conditions to avoid excessive seasonal BP change [45]. We plan to analyze the association between indoor temperature and seasonal BP variation in a future paper using BP data collected from optional surveys in summer.

### Interventions to retrofit houses with insulation and heating

**Fig. 5:**
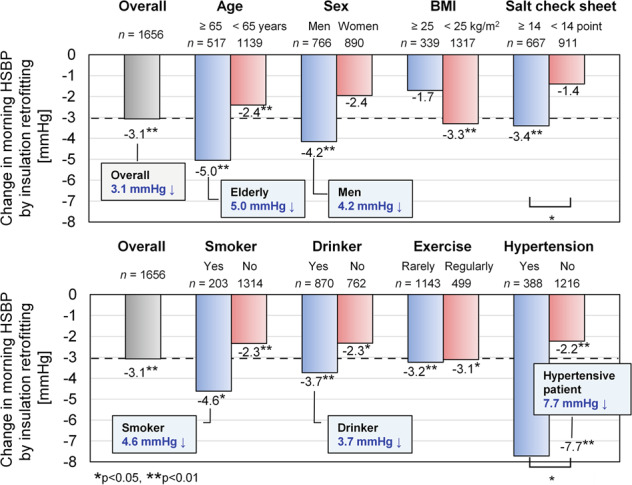
Change in morning home systolic blood pressure (HSBP) following insulation retrofitting by subgroup from ref. [45] †Each value shows the regression coefficient of multiple linear regression model adjusted for confounders. Blue bars indicate subgroups at high risk of cardiovascular diseases and red bars indicate subgroups at low risk of cardiovascular diseases

In the previous sections, we analyzed baseline data before insulation retrofitting. In this section, we present a longitudinal analysis of pre- and post-intervention data to clarify changes in HBP following insulation retrofitting by comparing HBP in the retrofitting and non-retrofitting groups [46]. Morning indoor temperature rose by 1.5 °C after insulation retrofitting, in spite of a slight decrease in outdoor temperature. Retrofitting insulation significantly reduced all four HBP indices (SBP and DBP in the morning and evening), for example morning SBP by 3.1 mmHg. Furthermore, we found a dose–response relationship between increased indoor temperature and decreased HBP, confirming that simple improvements in the thermal environment indoors can be effective. Another finding, shown in Fig. 5, was of greater heterogeneity in the effects of retrofitting insulation on morning SBP in self-reported hypertensive patients compared to normotensive occupants (–7.7 vs –2.2 mmHg). This indicates that the effects of insulation retrofitting were especially valuable in subgroups having a high risk of CVD.

A study of heating intervention and BP was conducted in Japan by Saeki et al. [47], in which physicians provided instructions to the participants on how to use their heating. This intervention on heating usage significantly decreased SBP and DBP by 4.4 and 2.3 mmHg, respectively, and the author recommended further research into the combination of insulation retrofitting and heating. A second intervention study combining insulation retrofitting and heating was conducted in Scotland by Lloyd et al. [48]. They examined improvements in BP produced by home renovation which included introduction of insulation material and central heating systems. The intervention significantly decreased SBP and DBP by 22 and 20 mmHg, respectively. Allowing that the sample size of this study was small, the combined effect of insulation retrofitting and heating appeared to greatly influence BP.

### Messages from the Smart Wellness Housing survey

We reviewed the effects of indoor temperature and insulation retrofitting on BP, focusing mainly on the national Smart Wellness Housing survey. In summary, we found: (1) about 90% of Japanese lived in cold homes (minimum indoor temperature <18 °C); (2) indoor temperature was nonlinearly associated with BP; (3) morning SBP was more sensitive to changes in indoor temperature than evening SBP; (4) SBP in older residents and women was particularly susceptible to indoor temperature change; (5) unstable indoor temperature was associated with large BP variability; and (6) insulation retrofitting intervention significantly reduced BP, especially in hypertensive patients.

**Fig. 6:**
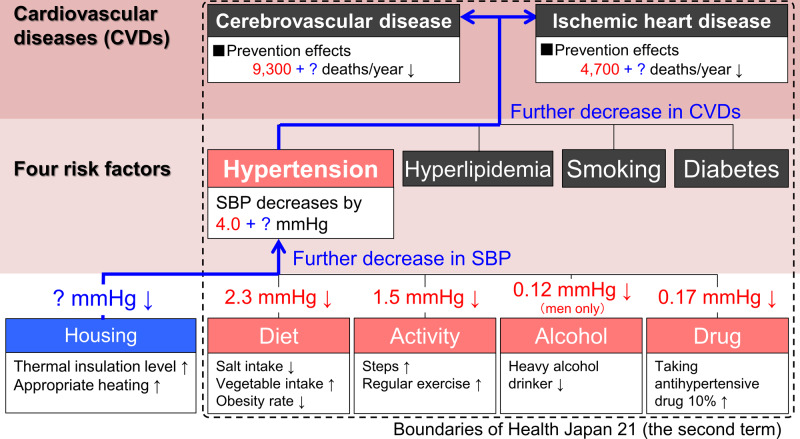
Expanded hierarchical structure for prevention of cardiovascular diseases including housing based on the present Japanese health policy “Health Japan 21 (the second term)” †Decreases in systolic blood pressure (SBP) by lifestyle modifications (e.g., 2.3 mmHg decrease by diet) and prevention effects on cardiovascular diseases (e.g., the prevention of 9,300 deaths/year due to cerebrovascular disease by 4.0 mmHg decrease in SBP) were quantified mainly based on epidemiological evidence

A systematic review [49] showed significant quantitative effects of lifestyle improvements including diet, aerobic exercise, alcohol and sodium restriction, as well as fish oil supplements on BP, namely mean reductions in SBP of 5.0, 4.6, 3.8, 3.6, and 2.3 mmHg, respectively. As described above, our study showed a significant morning SBP reduction of 3.1 mmHg following the insulation retrofitting of houses. Saeki et al. also showed SBP reduction effects of 4.4 mmHg by heating. Although evidence from housing environment interventions is scarce, we expect that the life-environment can exert a BP reduction effect comparable to that associated with lifestyle changes. At present, hypertension and CVDs are widely regarded as lifestyle diseases. However, we consider these diseases to be not only lifestyle diseases but also life-environment diseases, based on the SWH survey and previous research which proposed housing as one of the factors determining BP [50]. Our suggestion from this review is shown in Fig. 6. Although the present Japanese health policy [Health Japan 21 (the Second Term)] includes lifestyle factors only, such as diet, exercise, and alcohol consumption [51], “housing” should be included to further decrease BP and the number of deaths due to CVDs.

### The Smart Wellness Housing cohort survey

In today’s society, most people spend from 60% to 70% of their time at home [52–54]. It is therefore conceivable that our present findings on the short-term effect underestimate the effects of housing on BP, and so it is necessary to evaluate the long-term effect of housing on BP. We hypothesized that living in a cold home for more than 5 years had an adverse cumulative effect on cardiovascular health. We named this effect “cold debt”, with reference to sleep debt [55], which is a state of chronic sleep deprivation leading to physical and mental disorders.

In addition to the results presented in this review, the SWH survey revealed that the number of residents with high cholesterol was larger in colder homes [56]. A long-term risk of progression of arteriosclerosis is therefore present. We also found that a greater number of residents in colder homes had abnormal ECGs [57], which we assume to be a long-term consequence. In conclusion, as shown in Fig. 1, we started the Smart Wellness Housing cohort survey to clarify whether cold debt exists or not.

## Conclusion

The present review organizing evidence on housing and BP control indicated that housing has great potential for the prevention of hypertension in winter and the mitigation of EWM due to CVDs. We recommend life-environment improvements as well as lifestyle modifications to gain the additional benefit of decreasing patients with hypertension and CVDs. At present, as a target of life-environment improvements, 18 °C is widely accepted in general. However, it should be personalized depending on characteristics of residents because our evidence showed that vulnerability to indoor temperature was significantly different in each population group (e.g., age, gender). As a means of life-environment improvements, thermal insulation retrofit of houses was effective for a reduction in BP. We believe that the combination of insulation retrofit and appropriate heating use further enhance the importance of life-environment improvements in BP control.

## Supplementary information

Supplementary Information
