# Supplementary material for: Role of housing in blood pressure control: a review of evidence from the Smart Wellness Housing survey in Japan
Source: Hypertens Res. 2022 Oct 13;46(1):9–18. doi: 10.1038/s41440-022-01060-6 (PMC9747607; doi:10.1038/s41440-022-01060-6)
Supplement: Supplementary file 1 — Supplementary Information [file 41440_2022_1060_MOESM1_ESM.docx]

**Supplementary information**

Role of housing in blood pressure control: a review of evidence from the Smart Wellness Housing survey in Japan

Wataru Umishio,^1,2^ Toshiharu Ikaga,^2^ Kazuomi Kario,^3^ Yoshihisa Fujino,^4^ Masaru Suzuki,^5^ Shintaro Ando,^6^ Tanji Hoshi,^7^ Takesumi Yoshimura,^8^ Hiroshi Yoshino,^9^ and Shuzo Murakami^10^, on behalf of the SWH survey group

^1^ Department of Architecture and Building Engineering, School of Environment and Society, Tokyo Institute of Technology, Ookayama, Meguro-ku, Tokyo, Japan

^2^ Department of System Design Engineering, Faculty of Science and Technology, Keio University, Yokohama, Kanagawa, Japan

^3^ Department of Cardiology, Jichi Medical University School of Medicine, Shimotsuke, Tochigi, Japan

^4^ Department of Environmental Epidemiology, Institute of Industrial Ecological Sciences, University of Occupational and Environmental Health, Kitakyushu, Fukuoka, Japan

^5^ Department of Emergency Medicine, Ichikawa General Hospital, Tokyo Dental College, Ichikawa, Chiba, Japan

^6^ Department of Architecture, Faculty of Environmental Engineering, University of Kitakyushu, Kitakyushu, Fukuoka, Japan

^7^ Tokyo Metropolitan University, Hachioji, Tokyo, Japan

^8^ University of Occupational and Environmental Health, Kitakyushu, Fukuoka, Japan

^9^ Tohoku University, Sendai, Miyagi, Japan

^10^ Institute for Built Environment and Carbon Neutral for SDGs, Hirakawacho, Chiyoda-ku, Tokyo, Japan

Supplementary Table 1. Members of Smart Wellness Housing Survey Group

(a) Members of the Research Committee for the Promotion of Smart Wellness Housing

| **Chairperson** | |
| --- | --- |
| Shuzo MURAKAMI * | Institute for Building Environment and Energy Conservation |
| **Vice-chairperson** | |
| Takesumi YOSHIMURA * | University of Occupational and Environmental Health |
| Hiroshi YOSHINO * | Tohoku University |
| Kazuomi KARIO * | Jichi Medical University |
| **Organizer** | |
| Toshiharu IKAGA * | Keio University |
| **Committee member in medicine** | |
| Suminori AKIBA | Kagoshima University |
| Mikio ARITA | Sumiya Rehabilitation Hospital |
| Michiya IGASE | Ehime University |
| Masayoshi ICHIBA | Saga University |
| Nami IMAI | Mie University |
| Masaki UEMURA | At Home, LLC |
| Hiroyuki UEHARA | National Assembly Promoting Healthy and Energy Conserving Housing |
| Haruo UGUISU | Tokushima Bunri University |
| Kensuke ESATO | Yamaguchi University |
| Akira EBOSHIDA | Hiroshima University |
| Yuko OGUMA | Keio University |
| Toshiyuki OJIMA | Hamamatsu University School of Medicine |
| Shimato ONO | Marugame Ono Clinic |
| Yoshio OMATA | Hoju, Co., Ltd. |
| Takahiko KATOH | Kumamoto University |
| Masahiko KATO | Tottori University |
| Shinya KUNO | University of Tsukuba |
| Kiyokage KUBO | Kubo Clinic |
| Yoshiki KURODA | University of Miyazaki |
| Yasuaki SAIJO | Asahikawa Medical University |
| Kazuhiro SATO | University of Fukui |
| Eiji SHIBATA | Yokkaichi Nursing and Medical Care University |
| Kuninori SHIWAKU | Shimane University |
| Narufumi SUGANUMA | Kochi University |

(a) Members of the Research Committee for the Promotion of Smart Wellness Housing (continued)

| **Committee member in medicine (continued)** | |
| --- | --- |
| Tomotaka SOBUE | Osaka University |
| Toshiro TAKEZAKI | Kagoshima University |
| Masatoshi TANAKA | Fukushima Medical University |
| Tsuyoshi TANABE | Yamaguchi University |
| Susumu TSUKAMOTO | Saitama Jikei Hospital |
| Hiroyuki DOI | Okayama University |
| Kunio DOBASHI | Jobu Hospital for Respiratory Diseases |
| Chisato NAGATA | Gifu University |
| Hiroyuki NAKAMURA | Kanazawa University |
| Kunio NAKAYAMA | Former Osaka University |
| Norihiro NOGATA | Saiseikai Karatsu Hospital |
| Takashi HANATO | Eigenji Clinic |
| Yoshihisa FUJINO * | University of Occupational and Environmental Health |
| Tanji HOSHI * | Tokyo Metropolitan University |
| Satoshi HOSHIDE | Jichi Medical University |
| Takahiro MAEDA | Nagasaki University |
| Muneo MINOSHIMA | Minoshima Clinic |
| Takashi MURAWAKA | Yumemokuba, SNPC |
| Hidekazu YAMADA | Kindai University Nara Hospital |
| Misako YOSHINAGA | Kusunoki Hospital |
| **Committee member in architecture** | |
| Akihiko IWASA | Hosei University |
| Atsushi IWAMAE * | Kindai University |
| Akihito OZAKI | Kyushu University |
| Satoru KUNO | Nagoya University |
| Minoru KUMANO | Miyazaki University |
| Shoichi KOJIMA | Saga University |
| Yasuyuki SHIRAISHI | University of Kitakyushu |
| Hirotaka SUZUKI | Hokkaido Research Organization |
| Tsuyoshi SEIKE * | Tokyo University |
| Naoki TAKAGI | Shinshu University |
| Masaki TAJIMA | Kochi University of Technology |

(a) Members of the Research Committee for the Promotion of Smart Wellness Housing (continued)

| **Committee member in architecture (continued)** | |
| --- | --- |
| Yoshito TANAKA | Nagasaki Institute of Applied Science |
| Takayuki TAMAI | National Institute of Technology, Yonago College |
| Mitsutaka TSUJI | Gifu Academy of Forest Science and Culture |
| Reiji TOMIKU | Oita University |
| Hisaya NAGAI | Mie University |
| Daisaku NISHINA | Hiroshima University |
| Hideyo NIMIYA | Kagoshima University |
| Kenichi HASEGAWA | Akita Prefectural University |
| Hirofumi HAYAMA * | Hokkaido University |
| Akira FUKUSHIMA | Former Hokkaido University of Science |
| Yuji HORI | University of Toyama |
| Takeo MATSUOKA | Asia University |
| Teruaki MITAMURA | Maebashi Institute of Technology |
| Shinji YOSHIDA | Nara Women's University |

*: members of the Research Planning Committee for the Promotion of Smart Wellness Housing

(b) Members of the Subcommittee for Analysis of the Smart Wellness Housing Survey

| **Chairperson** | |
| --- | --- |
| Toshiharu IKAGA * | Keio University |
| **Vice-chairperson** | |
| Yoshihisa FUJINO * | University of Occupational and Environmental Health |
| **Organizer** | |
| Shintaro ANDO * | University of Kitakyushu |
| Tatsuhiko KUBO | Hiroshima University |
| **Committee member** | |
| Wataru UMISHIO | Tokyo Institute of Technology |
| Yuko OGUMA | Keio University |
| Naoki KAGI | Tokyo Institute of Technology |
| Hiroshi KANEGAE | Genki Plaza Medical Center for Health Care |
| Shun KAWAKUBO | Hosei University |
| Yoshinobu SAITO | Kanagawa University of Human Services |
| Keigo SAEKI | Nara Medical University |
| Masaru SUZUKI | Tokyo Dental College Ichikawa General Hospital |
| Tsuyoshi SEIKE * | Tokyo University |
| Takayuki TAJIMA | Tokyo Metropolitan University |
| **Experts committee member** | |
| Maki ITO | Japan Federation of Housing Organizations |
| Hiroshi KOJIMA | Keio University |
| Natsue DOIHARA | Keio University |
| **Adviser** | |
| Takesumi YOSHIMURA * | University of Occupational and Environmental Health |
| Kazuomi KARIO * | Jichi Medical University |
| Tanji HOSHI * | Tokyo Metropolitan University |

*: members of the Research Planning Committee for the Promotion of Smart Wellness Housing
